# Benefits of integrating eye care into health systems

**DOI:** 10.2471/BLT.18.221887

**Published:** 2018-10-01

**Authors:** Ivo Kocur, Etienne Krug, Silvio P Mariotti, Megan McCoy

**Affiliations:** a Management of Noncommunicable Diseases, Disability, Violence and Injury Prevention, World Health Organization, avenue Appia 20, 1211 Geneva 27, Switzerland.

Global estimates indicate that 217 million people have moderate to severe vision impairment and 37 million people are blind.^1^ Another 800 million people are estimated to have near vision impairment, that is, difficulty seeing up close, caused by presbyopia.^2^ As most vision impairment is avoidable, these estimates suggest that many eye-care service needs are not being met, such as cataract surgery or the provision of eye glasses to correct refractive error.

There have been many initiatives to prevent blindness and strengthen eye-care services. This includes efforts in the 1950s to prevent and control infectious diseases such as trachoma and onchocerciasis, to the development of broader national eye-care programmes across countries in the 1980s and the establishment of VISION2020^3^ for the elimination of avoidable blindness in the late 1990s.^4^ These initiatives have contributed to many achievements: effective interventions for many of the common causes of vision impairment have become available; data on the prevalence and causes of vision impairment have increased; and efforts to better monitor eye-care services have been made.^5^^–^^7^ Moreover, efforts to develop and support the eye-care workforce across ophthalmology, optometry and allied ophthalmic professions have taken place.^7^^,^^8^ These achievements make a solid foundation for the strengthening of eye-care services.

The increasing role of the sustainable development goals (SDGs) in driving national decision-making and changing population demographics provide a timely opportunity to consider the future of eye care, the topic for this theme issue.

The value of eye care, and therefore good vision, should be considered as part of the SDGs. Vision is particularly relevant to SDG 3 on good health and well-being, and to the target on universal health coverage, but also to other SDGs, such as those on education, gender equality, and decent work and economic growth. Countries should be able to make informed decisions, supported by evidence, when assessing the level of priority afforded to eye care and how to better integrate eye care into the health system.

Demographic changes, especially population growth and ageing, will increase the need for eye-care services,^1^ since older people have higher prevalence of eye diseases and greater risk of vision impairment. Furthermore, there are more people who need eye-care services than people with vision impairment: people with eye-care needs include those with health conditions such as diabetes and hypertension, which are often accompanied by vision-impairing complications, those who require systematic vision screening, such as infants and school-age children, and those with eye diseases that may not commonly lead to vision impairment, but that have painful and troublesome symptoms requiring care, such as dry eyes.

Responding to current and future population eye-care needs will require expanded access to quality services, not only for treatment but also for vision promotion and rehabilitation. More investments are needed to reduce eye disease incidence, curb the onset of vision impairment and to minimize the impact of disease. Rehabilitation for people with irreversible vision impairment is necessary to ensure improvement in their functioning and reduce disability.^9^

Increasing access to services will require renewed efforts to integrate eye care into the health system and into other sectors, such as education. There are many eye-care interventions that can be delivered through and with other health services, such as the prevention of retinopathy of prematurity through neonatal care^10^^,^^11^ and diabetic retinopathy through diabetes primary care.^11^ Vision screening can also be carried out through school health programmes.^12^ Integration is also critical across other areas of the health system, such as with the inclusion of key eye-care indicators in health information systems, provision of essential eye-care medicines to those in need, universal access to eye care and the integration of eye-care workforce planning within broader human resources for health plans.

This theme issue provides evidence of the value of integrating eye care across several areas, including training teachers in vision screening^12^ and the value of timely referrals for people with diabetes.^11^^,^^13^ Authors also discuss the importance of strengthening health information systems, including the need for making better use of prevalence data in strategic planning processes for eye care.^14^^,^^15^ The forthcoming World Health Organization’s *World report on vision* will provide countries with guidance on how to integrate eye care into the health system and will draw on the evidence, lessons learnt and perspectives presented here.
